# Estimating home-range size: when to include a third dimension?

**DOI:** 10.1002/ece3.590

**Published:** 2013-06-08

**Authors:** Pedro Monterroso, Neftalí Sillero, Luís Miguel Rosalino, Filipa Loureiro, Paulo Célio Alves

**Affiliations:** 1 CIBIO Centro de Investigação em Biodiversidade e Recursos Genéticos, Universidade do Porto, Inbio – Laboratório Associado, Campus Agrário de Vairão4485 – 661, Vairão, Portugal; 2 Departamento de Biologia, Faculdade de Ciências, Universidade do PortoRua do Campo Alegre s/n, 4150-150, Porto, Portugal; 3 Instituto de Investigación en Recursos Cinegéticos (IREC CSIC-UCLM-JCCM)Ronda de Toledo s/n, 13005, Ciudad Real, Spain; 4 Centro de Investigação em Ciências Geo-Espaciais (CICGE), Universidade do Porto, Faculdade de Ciências, Observatório Astronómico Prof. Manuel de BarrosAlameda do Monte da Virgem 4430-146, Vila Nova de Gaia, 4169-007, Porto, Portugal; 5 Universidade de Lisboa, Centro de Biologia Ambiental, Faculdade de Ciências de LisboaEd. C2 Campo Grande, 1749-016, Lisboa, Portugal; 6 Laboratório de Ecologia Isotópica/CENA/Universidade de São PauloPO Box 96, Piracicaba, São Paulo, 13416-000, Brasil; 7 University of Montana Wildlife Biology Program, College of Forestry and Conservation32 Campus Drive, Missoula, Montana, 59801

**Keywords:** Mammalian ecology, modeling, planimetric home-range, slope threshold, topographic home-range

## Abstract

Most studies dealing with home ranges consider the study areas as if they were totally flat, working only in two dimensions, when in reality they are irregular surfaces displayed in three dimensions. By disregarding the third dimension (i.e., topography), the size of home ranges underestimates the surface actually occupied by the animal, potentially leading to misinterpretations of the animals' ecological needs. We explored the influence of considering the third dimension in the estimation of home-range size by modeling the variation between the planimetric and topographic estimates at several spatial scales. Our results revealed that planimetric approaches underestimate home-range size estimations, which range from nearly zero up to 22%. The difference between planimetric and topographic estimates of home-ranges sizes produced highly robust models using the average slope as the sole independent factor. Moreover, our models suggest that planimetric estimates in areas with an average slope of 16.3° (±0.4) or more will incur in errors ≥5%. Alternatively, the altitudinal range can be used as an indicator of the need to include topography in home-range estimates. Our results confirmed that home-range estimates could be significantly biased when topography is disregarded. We suggest that study areas where home-range studies will be performed should firstly be scoped for its altitudinal range, which can serve as an indicator for the need for posterior use of average slope values to model the surface area used and/or available for the studied animals.

## Introduction

The home-range of an animal is traditionally defined as “the area traversed by the individual in its normal activities of food gathering, mating, and caring for young” (Burt 1943). This concept has suffered some adjustments over time (e.g., Hayne 1949; Harris et al. 1990) nevertheless it remains as a geographically explicit area used by an individual. Among the characteristics of a home-range, three are particularly important: size, shape, and structure (Kenward 2001). The assessment of these features is generally needed for understanding the biological requirements of a species or population, intra- and interspecific relations and allowing for subsequent management actions, such as the design of reserves (e.g., Biebouw 2009).

Home-range size is affected by several factors such as animal size, metabolic needs, resource availability, and population density (Cooper 1978; Gittleman and Harvey 1982; Lindstedt et al. 1986; Litvaitis and Sherburne 1986). However, other factors, such as topography, may constrain the size and shape of a specific home-range, for example, by making its limits coincide with particular topographic features (Reid and Weatherhead 1988; Powell and Mitchell 1998). Nevertheless, the importance of topography in the determination of home-range size and shape is often disregarded, not only as a potential factor shaping the spatial placement and configuration of individual home ranges within a population, but also as a feature under which the actual area occupied by an animal is supported. Most studies dealing with home ranges consider the study areas as if they were totally flat (i.e., planimetric), working only in two dimensions (e.g., Broomhall et al. 2003; Molina-Vacas et al. 2009). However, the effectively available area for the animals is an irregular surface, displayed in three dimensions (i.e., including topography) (Stone et al. 1997; Campbell et al. 2004; Greenberg and McClintock 2008). By ignoring the third dimension, as many studies do (e.g., Admasu et al. 2004; Finďo and Chovancová 2004; Simcharoen et al. 2008; Mattisson et al. 2011), the size of home ranges is expected to underestimate the surface actually occupied by the animal. Moreover, the severity and importance of these underestimations should be related to the roughness of the terrain, and thus the differences between the planimetric and the topographic areas are expected to be larger in mountainous or highly irregular terrains than in smoother surfaces characterized by gentle slopes. Topographic home ranges, that is, the home-range considering its topographic surface, have been less often explored by wildlife researchers (e.g., Geffen et al. 1992; Powell and Mitchell 1998; Campbell et al. 2004; Greenberg and McClintock 2008). Some of these studies detected a variety of differences between the planimetric and topographic home-range estimates: 3.1% for white-tailed deers (*Odocoileus virginianus*; Campbell et al. 2004); 6.4% for allegheny woodrats (*Neotoma magister*; Castleberry et al. 2001); 9% for diamond rattlesnakes (*Crotalus ruber*; Greenberg and McClintock 2008); 14% for speckled rattle snakes (*Crotalus mitchellii*; Greenberg and McClintock 2008); 20% for yaku monkeys (*Macaca fuscata*; Sprague 2000); and 23% for black-and-white snub-nosed monkeys (*Rhinopithecus bieti*; Grueter et al. 2008). For these reasons, and as topography can affect the animal's perception of the habitat, food resources, or access to mates (Powell and Mitchell 1998), ignoring this factor can lead researchers to misinterpret the ecological needs of animals, mainly if they inhabit mountainous or rough areas. Apart from the implications on individual home-range estimates, ignoring topography may also affect the interpretation of other ecological parameters. One example is illustrated with yaku monkeys, whose travel distances estimates increase in 10% if terrain roughness is considered (Sprague 2000). The adequate interpretation of such parameters is relevant, for instance, when analyzing the energy costs associated with patrolling and scent-marking activities. This may be particularly important when dealing with carnivores, whose spatial scale of landscape use and selection is large enough to be affected by topography variation, resulting from the projected surface area relation (Dickson and Beier 2007). Moreover, carnivores generally rest and breed in natural vegetated roughed areas, where they feel safe from human disturbance (e.g., Powell and Mitchell 1998; Rosalino et al. 2004; Monterroso et al. 2009). As several carnivore species inhabit mountainous regions (e.g., Nilsson and Götmark 1992; Powell et al. 2000), taking into account terrain roughness assumes a fundamental role in management and conservation planning.

Despite the recognized importance of including topography in the estimate of home-range sizes, and the suggestion of some authors that this variable should always be considered in home-range estimation (Greenberg and McClintock 2008), this might not be justified in areas with smooth orography where the variation between planimetric and topographic home-range estimates might not be significant. On the other hand, and even though Geographic Information Systems' (GIS) tools are currently available and accessible to nonexperts for a wide range of spatial analysis, in some cases, terrain modeling can be a challenging task.

Furthermore, producing topographic estimates may often be a time- and money-consuming task, which might not compensate, especially if the difference between planimetric and topographic areas is not significant. However, to our knowledge, no simple and robust method is currently available to evaluate the necessity of using topographic home-range estimates, or if by using planimetric estimates researchers are not incurring in significant errors. Therefore, we hypothesize that the variation between planimetric and topographic estimates can be modeled using terrain characteristics as potential explanatory factors. In this context, our objective was to analyze the influence of considering a third dimension in the estimation of home-range size. Using simulated data, we modeled the variation between the planimetric and topographic home-range estimates considering different topographic features as potential explanatory variables at several spatial scales. As final output, we provide general rules to decide when topographic estimates should be used instead of the traditional planimetric home-range ones, through a simple landscape evaluation of the study area.

## Materials and Methods

### Area selection

**Figure 1:**
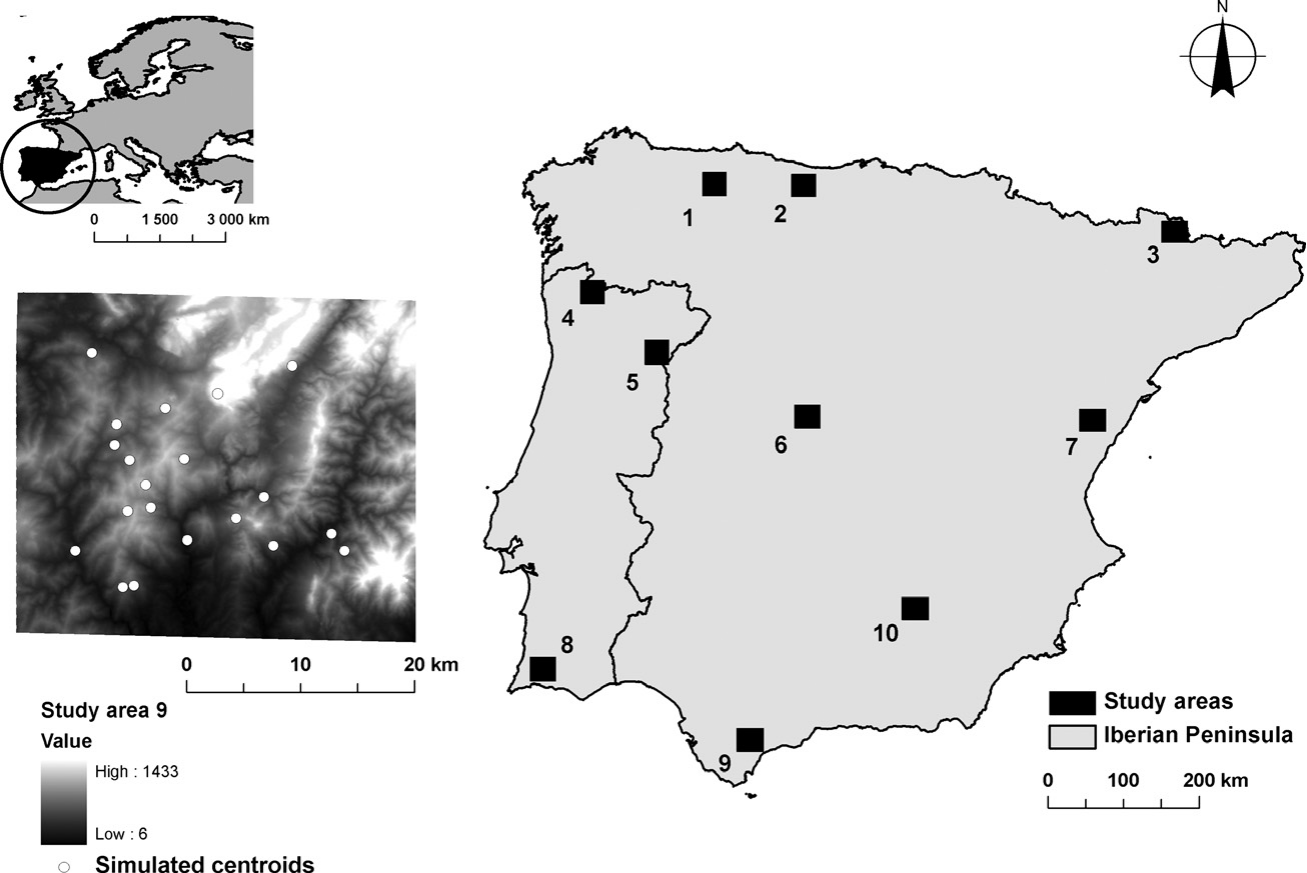
Distribution of the study areas in the Iberian Peninsula and example of the simulated sampling scheme within the study areas.

The Iberian Peninsula (IP) was considered the spatial unit for our simulations, under which potential study areas were selected (Fig. 1). The overall criteria for selecting the study areas were (i) widely dispersed through the IP and (ii) marked topographic variation upon visual analysis. Under these criteria, 10 study areas were selected across the entire IP, upon a preliminary assessment of their orography. Each study area was a polygon with dimensions of 0.27 latitudinal degrees and 0.40 longitudinal degrees, roughly 900 km^2^ (ca 30 × 30 km).

### Spatial data layers

#### Elevation data and derived variables

Elevation data for the 10 areas were obtained from the ASTER (Advanced Spaceborne Thermal Emission and Reflection radiometer) global digital elevation model (GDEM: http://www.gdem.aster.ersdac.or.jp), which covers the Earth's land surface between 83°N and 83°S latitudes with a spatial resolution of 30 m. It was produced by Japan-US ASTER Science Team using ASTER data acquired from the start of observation until the end of August 2008. We clipped the GDEM files by the 10 areas, and derived the following three variables: planimetric area, topographic area, and slope. The planimetric area represents the flat area of a region, without considering its topographic surface, and therefore equals the area of the trapezoidal boundary of that region of interest. The planimetric surfaces were calculated with the ArcView 3.2's (ESRI 2000) extension “Surface Tools v.1.6b” (Jenness 2008) by summing the areas of the pixels comprised within the area of interest.

The topographic area represents an approximation to the real surface area of a region, and consequently always will be larger or equal to planimetric area. We derived the topographic surface using Jenness's (2004) method, with the ArcView 3.2's (ESRI 2000) extension “Surface Tools v.1.6b” (Jenness 2008), now available for ArcGIS 10 (Jenness 2013). The surface area of a particular pixel on a digital elevation model is calculated by the sum of triangle areas derived from eight triangles using the eight surrounding pixels. In each of these eight pixels, a center point is created and connected with the center point of the central cell (the pixel situated in the middle of the eight pixels) and with the center points of two adjacent cells, in order to create a triangle. These triangles are located in three-dimensional space, and the center points are placed in their respective elevations. Therefore, the triangle surface represents the topographic surface. In order to obtain the area of the triangle surfaces, the extension “Surface Tools v.1.6b” (Jenness 2008) calculates the lengths of the eight lines connecting the central point of the center cell with the center points of the eight surrounding cells, as well as the lengths of the lines connecting each of the eight surrounding cells with the one right next to it. The result is the lengths of the sides of eight triangles meeting at the center point of the central cell. The area of the triangles is calculated using the Pythagorean Theorem [1]:


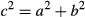
(1)

where *a* is the planimetric distance between two center points; *b* is the elevation difference between two center points; and *c* is the true surface distance between these two center points. *A* is simply the value of the cell size for the cells directly to the North, East, South, and West. For the diagonals, using again the Pythagorean Theorem, *a* values:


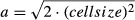
(2)

The extension calculates the total area of each triangle using the Triangle half-perimeter formula. The triangle half-perimeter (S) is calculated as


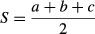
(3)

and the area (A) as


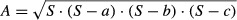
(4)

Therefore, it is necessary to previously clip all the triangle lengths in half. This new clipped triangle is similar to its corresponding original triangle because the two sides extending from the center cell are exactly the half in length to the respective sides in the original triangle, and the angles defined by these two sides are the same in each triangle. Thus, the third side of the clipped triangle must be exactly half in length to the corresponding side of the original triangle (Jenness 2004).

Finally, slope was derived from the ASTER GDEM using ArcGIS 9.2's extension “Spatial Analyst” (ESRI 2007). The slope function calculates the maximum rate of change between each cell and its neighbors; for example, the steepest downhill descent for the cell (the maximum change in elevation over the distance between the cell and its eight neighbors; ESRI 2007). The slopes' average and standard deviation were calculated for all regions of interest.

### Data simulation

**Figure 2:**
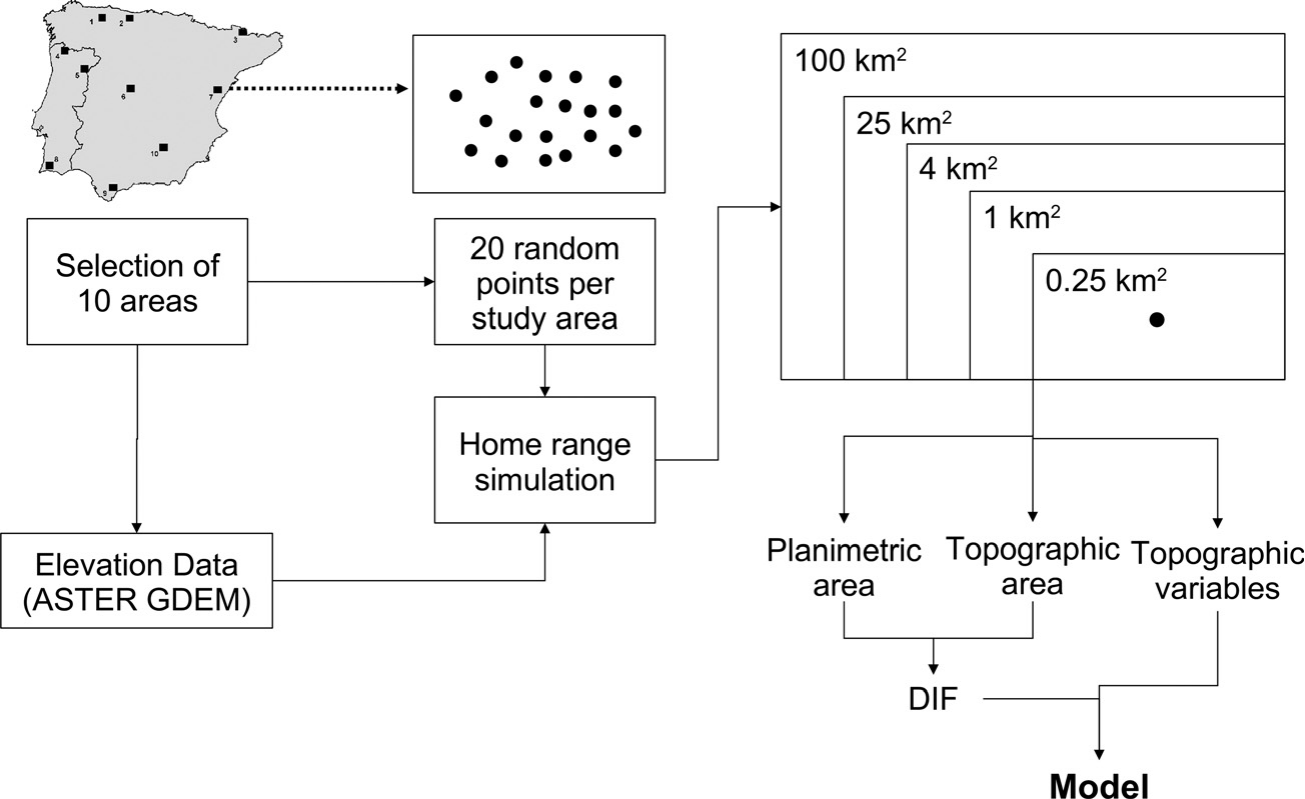
Flowchart illustrating the simulation of home ranges within each study area, and the extraction of ancillary variables.

Within each study area, 20 randomly distributed points were generated using Hawths' tools v.3.4 extension (Beyer 2004) for ArcGIS 9.2 software. Therefore, 200 points were created in total. Point location within each study area was forced to be excluded from a 10-km radius of the external border in order to prevent bias in the subsequent spatial analysis. Each generated point constituted the centroid of simulated home ranges, which consisted of flat square areas of 100, 25, 4, 1, and 0.25 km^2^ around it (Fig. 2). The different home-range areas pretend to simulate different scales of analysis, as different species tend to require different home-range sizes to fulfill their metabolic needs. The simulated home ranges encompassed a wide range of topographic characteristics with average altitude ranging from less than 100 m to over 2300 m a.s.l. (Table 1). Average slopes were of approximately 16°, and ranged from 1° up to more than 35° (Table 2). For each simulated home-range, the total planimetric and topographic areas were calculated as well as the altitudinal range, slope and altitude average, and correspondent standard deviations. There are several approaches to home-range estimation. Some are polygon based (e.g., the minimum convex polygon – MCP), others are based on grid-cell counts (e.g., Siniff and Tester grid method) or can even be probabilistic methods (e.g., kernel methods; Millspaugh and Marzluff 2001). To compare estimates of planimetric and topographic home ranges, we used an adaptation of the MCP method using square areas. We acknowledge that different estimators, when applied to the same data, may result in different home range's shapes and sizes. Regardless, for the purpose of testing the differences between planimetric and topographic home ranges, the main issue is that the analytical method should be maintained constant so that results may be comparable.

The difference between planimetric and topographic areas was calculated as the percent difference (Stone et al. 1997; Campbell et al. 2004):


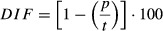


where *DIF* is the percent difference between the planimetric and the topographic areas, *p* is the planimetric area, and *t* is topographic area of each simulated home-range. Following Campbell et al. (2004) suggestion, we considered 5% as the threshold for considering *DIF* values as relevant for topography to be accounted for in home-range calculations. Nevertheless, in order to present more conservative approaches, *DIF* values of 10, 20, and 30% will also be considered in this study.

### Modeling the topographic–planimetric difference

The determinants of the percent difference between planimetric and topographic areas (hereafter *DIF*) were evaluated using linear regression analysis (Sokal and Rohlf 1995). The average altitude (AVG_ALT), altitude standard deviation (SD_ALT), altitudinal range (RANGE_ALT), average slope (AVG_SLP), and slope standard deviation (SD_SLP) were explored as potential explanatory variables. All variables were checked for colinearity as it may inflate p-values, thus making it more difficult to detect significant effects (Zuur et al. 2010). All variables were log transformed in order to achieve normality and homogeneity of variance (Zuur et al. 2010). Simple linear regressions were performed in order to evaluate variable's individual effect on *DIF*. The colinearity found between potential explanatory variables prevented the use of multiple linear regression analysis.

Model fit was assessed using *R*^2^, as defined by Kvalseth (1985). Using the linear regression models, we selected the variable that produced the more stable linear models (the average slope – see Results) to estimate the values needed to achieve *DIF* values of 5, 10, 20, and 30%.

In practical works, digital information on the study areas may not be available to researchers (however, see Sillero and Tarroso 2010), and only basic topographic features, such as minimum and maximum elevation, are known. In order to evaluate if the altitudinal range could be used as an indicative variable of a potential value range for *DIF*, we plotted the *DIF* values obtained from the different simulated home ranges within each study area against their altitudinal difference (defined as the difference between the maximum and the minimum elevations in the study area). To facilitate the graphical interpretation of the results, study areas were grouped in 200-m altitudinal difference intervals. A Student's *t*-test was used to investigate the significance of the difference in *DIF* estimates between study areas with altitudinal ranges smaller and higher than 1800 m. All analyses were performed using the software Statistica v7.1 (Statsoft, Inc 2005). Unless otherwise referred, all values are presented as average ± standard deviation.

## Results

The selected study areas covered a wide range of topographic characteristics. Average altitudes ranged from 186 m to 1758 m a.s.l. (Table 1). Moreover, the altitudinal ranges within each study area varied from 888 m to 2522 m (Table 1). Five of the study areas (1, 2, 3, 4, and 7) are included in the Atlantic bioclimatic region of the IP (Rivas-Martínez et al. 2004), where average temperature ranges from 0.8 ± 3.5°C to 23.9 ± 2.5°C (Hijmans et al. 2005). Four others (5, 8, 9, and 10) are located in the Mediterranean Bioclimatic region, where ambient temperature often rises above 35°C during the warmer seasons (Rivas-Martínez et al. 2004; Hijmans et al. 2005). Study area 6 is located in a transition zone between the Mediterranean and Atlantic regions.

**Table 1:** Topographic characteristics of the 10 study areas

Area ID	Country	Longitude	Latitude	Altitude (m)				

				Min.	Max.	Average	SD	Range
1	Spain	6.2011°W	43.1638°N	148	2100	1055	405	1952
2	Spain	4.7510°W	43.1788°N	54	2576	1129	511	2522
3	Spain	1.2390°E	42.5639°N	703	3077	1758	454	2374
4	Portugal	8.0809°W	41.8139°N	55	1513	795	281	1458
5	Portugal	7.0111°W	41.1389°N	−14	901	415	170	915
6	Spain	4.6210°W	40.4337°N	532	1991	1083	286	1459
7	Spain	0.1809°W	40.3637°N	298	1750	937	288	1452
8	Portugal	8.5008°W	37.3037°N	4	892	186	134	888
9	Spain	5.3811°W	36.5638°N	6	1433	511	270	1427
10	Spain	2.9410°W	38.1537°N	385	1923	827	291	1538

Longitude/Latitude – location of the study area centroid. Coordinates in WGS84 geographic system.

Topography varied across the different simulated home ranges, as reflected in the *DIF* values which ranged from nearly zero to more than 20% (Table 2). The average *DIF* values found across all simulated home ranges were above 5% in all spatial scales.

**Table 2:** Topographic characteristics of the simulated home ranges at 100, 25, 4, 1, and 0.25 km^2^ scales (data are presented as mean value ± standard deviation and variation range)

Scale (km^2^)	Altitude (m)	Slope (°)	*DIF* (%)
100	874 ± 465 (90–2352)	16.62 ± 6.11 (6.77–33.19)	6.03 ± 4.00 (1.05–21.42)
25	853 ± 488 (63–2274)	16.39 ± 6.41 (5.40–26.08)	5.95 ± 4.12 (0.65–18.86)
4	851 ± 503 (34–2319)	16.62 ± 6.81 (3.79–36.07)	6.02 ± 4.43 (0.36–22.13)
2	852 ± 507 (27–2355)	16.58 ± 7.44 (1.64–35.34)	5.94 ± 4.75 (0.11–19.35)
0.25	852 ± 508 (27–2370)	16.41 ± 7.77 (1.00–38.50)	5.74 ± 21.96 (0.04–22.00)

DIF, percent difference in area size between planimetric and topographic estimates.

**Figure 3:**
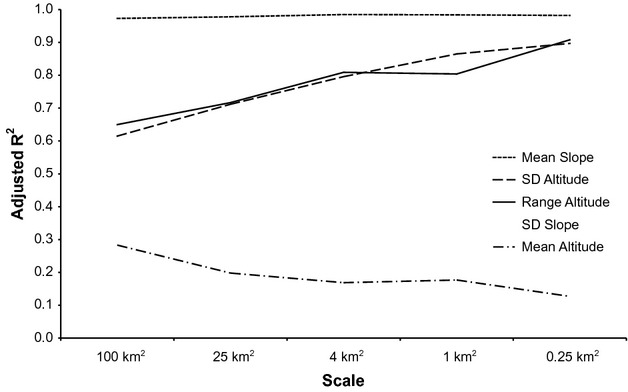
Variation in linear models' (difference in area between planimetric and topographic surfaces versus independent variables) fit across the different scales of analysis. Mean slope, Mean slope; SD Altitude, Altitude standard deviation; Range Altitude, Altitudinal range; SD Slope, Slope standard deviation; Mean Altitude, Mean altitude.

Linear regression analysis between log-transformed values of *DIF* and AVG_SLP produced very good models for all home-range sizes, with average *R*^2^ values of 0.980 ± 0.005 (Table 3; Fig. 3).

**Table 3:** Linear regressions between log-transformed values of *DIF* (percent difference in area size between planimetric and topographic estimates) and AVG_SLP, at 100, 25, 4, 1, and 0.25 km^2^ home ranges

Area (km^2^)	Intercept	Slope coefficient (β)	Adj. *R*^2^	SE of estimate	*P*-value
100	−1.476	1.813	0.973	0.048	<0.001
25	−1.509	1.839	0.978	0.050	<0.001
4	−1.596	1.896	0.985	0.047	<0.001
1	−1.601	1.887	0.984	0.056	<0.001
0.25	−1.614	1.885	0.982	0.062	<0.001

**Figure 4:**
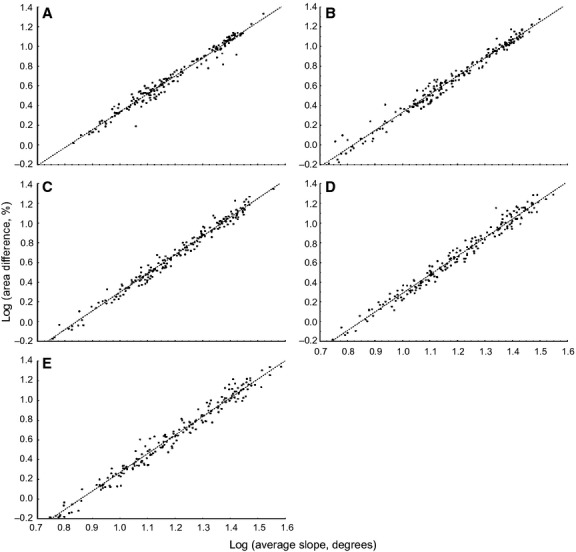
Linear regression plots of difference between planimetric and topographic surfaces (DIF) versus average slope at different scales of analysis: a) 100 km^2^; b) 25 km^2^; c) 4 km^2^; d) 1 km^2^; e) 0.25 km^2^.

We found no effect of home-range scale on model fitting, (Table 3), as all models had similar slopes (1.864 ± 0.036) and intercept values (−1.559 ± 0.062). Moreover, AVG_SLP was the variable that produced the more stable linear models across different spatial scales, as the values of the adjusted *R*^2^ were always maintained above 0.97 (Table 3; Fig. 4). AVG_ALT revealed to be a poor predictor of *DIF*, whereas the SD_SLP provided unstable *R*^2^ values in the models across spatial scales (Appendix S1).

According to our models, an average slope of approximately 16.3^o^ (±0.4) would be needed to attain 5% difference between planimetric and topographic home-range areas at all home-range sizes (Table 4). In 40.93, 43.50, 48.50, 45.0, and 45.0% of the home ranges simulated at 100, 25, 4, 1, and 0.25 km^2^ scales, respectively, this threshold value was reached. The average slope needed to achieve a 10% of difference was 23.6^o^ (±0.5). The 10% threshold was achieved in 20.7, 19.5, 19.5, 22.5, and 22.0% of the simulated 100, 25, 4, 1, and 0.25 km^2^ home ranges, respectively. Average slopes of 34.2 ^o^ (±0.6) and 42.5 (±0.7) would be needed to achieve 20% and 30% *DIF* values, respectively.

**Table 4:** Predicted average slope (in degrees) threshold to obtain *DIF* (percent difference in area size between planimetric and topographic estimates) values of 5, 10, 20, and 30%

Area (km^2^)	Predicted area difference (*DIF*)			

	5%	10%	20%	30%
100	15.83	23.21	34.01	42.53
25	15.86	23.12	33.70	42.01
4	16.24	23.41	33.74	41.79
1	16.54	23.88	34.48	42.74
0.25	16.86	24.35	35.17	43.62
Average slope	16.27	23.59	34.22	42.54
SD	0.44	0.51	0.62	0.72

**Figure 5:**
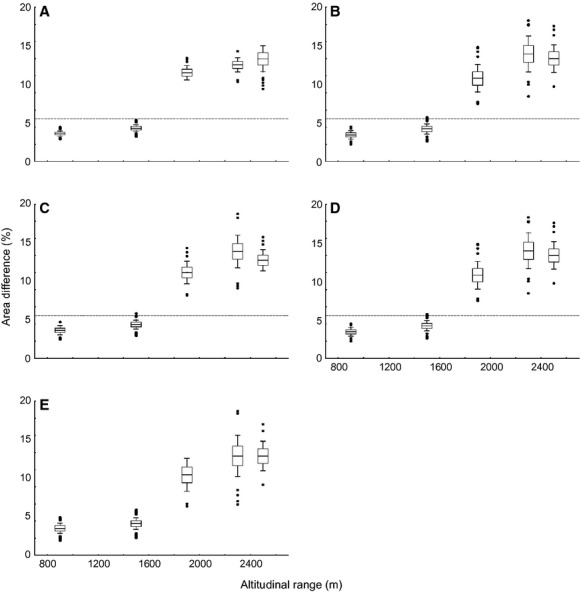
Average area difference between planimetric and topographic surfaces in relation to the altitudinal range at different scales of analysis: a) 100 km^2^; b) 25 km^2^; c) 4 km^2^; d) 1 km^2^; e) 0.25 km^2^.

The visual analysis of the *DIF* versus altitudinal range plots revealed that, despite the high variability observed in each altitudinal range class, study areas that had altitudinal ranges above the 1800 m tend to have significant *DIF* values, that is, above 5% (Fig. 5). This is corroborated by the statistical difference between *DIF* values of the areas above and below 1800 m. The average *DIF* values for home ranges in study areas with altitudinal ranges beneath and above 1800 m were of 1.30 and 5.74%, respectively. The t-test result for these two groups revealed that they were statistically different at all spatial scales (*P* < 0.01 for all scales; see Appendix S2).

## Discussion

Our results revealed that home-range size estimations based on planimetric approaches often produce underestimated measurements, especially in areas with adverse orography. Moreover, we managed to develop a simple and robust technique to assess the need to consider topography in home-range estimation based on the study area's average slope.

Topography influences the studies on the ecology of terrestrial animals at two different levels. On one hand, it has a direct influence on the animals' ecology, by constraining their home-range spatial configuration, as they may have the need to adapt its boundaries, shape, and use in face of the spatial location of topographic elements (Reid and Weatherhead 1988). For example, when present, ridges usually constitute territory limits for Ipswich sparrows (*Passerculus sandwichensis*) as they obstruct the view over the home-range, leading to an increase in defense costs of those ridge areas, and the consequence exclusion from the animals' territories (Reid and Weatherhead 1988). In this context, topography is an environmental factor, which might be of high importance for animals' biological processes and ecological strategies, as recognized in several studies (e.g., Sprague 2000; Campbell et al. 2004; Fan and Jiang 2008). However, the topographic effects are often neglected by researchers, especially at fine-scale analysis. This second level of influence constrains researchers' perception of animals' ecological processes. Many researchers recognize topographic factors as determinant at large-scale studies, such as distribution modeling, and often incorporate them in species distributions models (e.g., Kavanagh and Stanton 2005; Monterroso et al. 2009; Real et al. 2009). At finer scales, such as population or individual levels (e.g., home-range estimations and habitat selection assessments), although the topographic effect may seem less important, the consequence of ignoring it may be severe in terms of conservation actions and population management strategies. The method proposed in this study can help researchers decide whether to consider a third dimension (topography) in such spatial analysis, and shows that studies implemented in areas with an average slope greater than 16.3°, or with an altitudinal range wider than 1800 m, should consider the topographic structure of the area when defining individual home ranges. Above these thresholds, the difference between estimated planimetric and topographic home-range areas is likely to exceed 5%.

As mentioned above, among all topography-related variables analyzed, average slope showed the strongest relationship with the difference between planimetric and topographic areas, and the most stable correlation with the difference between both surfaces, being suitable to use at all spatial scales (Fig. 3). This was not an unexpected result as the greater the surface area with wide angles (with the horizontal plane), the greater is the expected difference between its projected and real area. Altitude-related variables (such as maximum altitude and altitudinal range) correlated less with the independent variable than slope-related ones. High altitudes do not necessarily suppose orographic variability (e.g., a high plateau). Even a wide altitudinal range may occur in an area dominated by gentle slopes, where a single topographic structure occurs (e.g., a canyon).

The close relationship between the average slope and the topographic and planimetric area difference is maintained across the spatial scales considered (Fig. 3). This pattern was not detected for the other variables, as variability tends to decrease with spatial scale reduction. Our results also suggest that, if slope data are not available or if is impossible to be derived for a study area, altitudinal range and standard deviation of altitude should preferably be used, but only if the target species' home-range is small (below 4 km^2^; Fig. 3).

Despite our general suggestion that disregarding topography may lead to bias in home-range studies, we are aware that some species are more affected by this factor than others. Species which are often associated with mountainous areas, in low densities, sparse distribution, and that have elusive behavior are obviously more subject to these errors. Within the mammalian fauna of the IP (where our simulation areas were selected), such species are, for example, the chamois (*Rupicapra rupicapra*), the Spanish ibex (*Capra pyrenaica*), or the brown bear (*Ursus arctos*). For instance, the study of Quenette et al. (2001) on brown bears spatial ecology in the French Pyrenees was implemented in an area with an altitudinal range of nearly 2400 m. Upon a simple evaluation of the Figure 5, we would expect that the topographic home-range of the studied animal to be underestimated by at least 10%. The same argument could be extended to Palomero et al. (1997) study of another population of brown bears (in the Cantabrian Mountains). In this case, an altitudinal range of 1800 m suggests that home-range estimates might have been underestimated by 8–10%. At a global scale, another potential good example is snow leopards (*Panthera uncia*), an endangered species whose preferential habitat is located in high altitudes with steep slopes, ranging from 3600 to 6000 m a.s.l. (Oli 1997). In such an area, with an altitudinal range of more than 2000 m, the estimated animals' home-range might be 10% smaller than the real area. If home ranges are used as a surrogate of this predator density, this population parameter will be overestimated, inducing researchers to consider that the status of this population is better than it really is.

Although the impact of ignoring topography in home-range estimation studies has already been highlighted by other authors, the decision of including this third dimension in analysis remains a researchers' option. Frequently, home-range estimations are based in a two-dimensional world and the consequences of doing so are overlooked. Our study suggests a set of guidelines that support researchers in the decision of whether to include topographic corrections based on the potential errors produced by a simple 2D analysis. We provide two simple threshold assessment methods to decide if topographic surfaces should be incorporated into home-range estimates. Furthermore, given the variability in study areas included in this study and within study areas simulated home ranges, we believe that the presented models should allow their applicability by wildlife researchers worldwide and to a large variety of species. Currently, altitudinal data as well as free/open source GIS software are unreservedly available (mainly through Internet; Sillero and Tarroso 2010), providing opportunities for the application of our proposed methodology. By applying it, researchers may save precious time that may be devoted to other analysis, without compromising the study validity or quality. Other researchers may also use it to easily validate the accuracy of density estimations based on home ranges, published in conservation studies and plans. Nevertheless, we suggest that similar studies should be conducted in other mountainous regions of the world in order to verify if the thresholds values and models found in this study remain constant. We hypothesize that threshold values should be similar in other study areas, as we performed the analysis over very different topographic areas using a method independent of artificial considerations (e.g., administrative limits). Further research should also be directed to the impacts of ignoring topography in other aspects of animal ecology and physiology, such as distance costs, energetic requirements or habitat selection, and consequently, in management decisions.

## Supporting Information

**Appendix S1.** Linear regressions' summary between log-transformed *DIF* values and the independent variables.

**Appendix S2.** *T*-tests results for the *DIF* values for areas with altitudinal ranges beneath and above 1800 m.
