# Supplementary material for: Estimating home-range size: when to include a third dimension?
Source: Ecol Evol. 2013 Jun 8;3(7):2285–95. doi: 10.1002/ece3.590 (PMC3728965; doi:10.1002/ece3.590)
Supplement: Supplementary file 1 [file ece30003-2285-SD1.doc]

**Appendix S1**. Linear regressions’ summary between log-transformed *DIF* values and each of the considered independent variables, at 100km^2^, 25km^2^, 4km^2^, 1km^2^ and .25km^2^ home-ranges.

| **Area size** | **Independent variable** | **Intercept** | **Slope coefficient (β)** | **Adj. R^2^** | **St. Err. of estimate** | **p-value** |
| --- | --- | --- | --- | --- | --- | --- |
| **100km^2^** | **Log_10_(avrg_alt)** | -0.920 | 0.560 | 0.283 | 0.252 | <0.001 |
|  | **Log_10_(sd_alt)** | -1.738 | 1.064 | 0.615 | 0.184 | <0.001 |
|  | **Log_10_(range_alt)** | -2.843 | 1.189 | 0.650 | 0.176 | <0.001 |
|  | **Log_10_(sd_slope)** | -1.639 | 2.554 | 0.739 | 0.152 | <0.001 |
| **25km^2^** | **Log_10_(avrg_alt)** | -0.743 | 0.493 | 0.198 | 0.305 | <0.001 |
|  | **Log_10_(sd_alt)** | -1.819 | 1.158 | 0.712 | 0.183 | <0.001 |
|  | **Log_10_(range_alt)** | -2.895 | 1.266 | 0.717 | 0.181 | <0.001 |
|  | **Log_10_(sd_slope)** | -0.425 | 1.392 | 0.606 | 0.213 | <0.001 |
| **4km^2^** | **Log_10_(avrg_alt)** | -0.740 | 0.487 | 0.169 | 0.353 | <0.001 |
|  | **Log_10_(sd_alt)** | -1.846 | 1.294 | 0.796 | 0.175 | <0.001 |
|  | **Log_10_(range_alt)** | -3.017 | 1.420 | 0.809 | 0.169 | <0.001 |
|  | **Log_10_(sd_slope)** | -1.137 | 2.125 | 0.820 | 0.164 | <0.001 |
| **1km^2^** | **Log_10_(avrg_alt)** | -0.944 | 0.547 | 0.177 | 0.396 | <0.001 |
|  | **Log_10_(sd_alt)** | -1.889 | 1.468 | 0.865 | 0.160 | <0.001 |
|  | **Log_10_(range_alt)** | -2.493 | 1.294 | 0.804 | 0.193 | <0.001 |
|  | **Log_10_(sd_slope)** | -1.001 | 2.057 | 0.802 | 0.194 | <0.001 |
| **0.25km^2^** | **Log_10_(avrg_alt)** | -0.846 | 0.499 | 0.127 | 0.436 | <0.001 |
|  | **Log_10_(sd_alt)** | -1.708 | 1.550 | 0.897 | 0.150 | <0.001 |
|  | **Log_10_(range_alt)** | -2.781 | 1.605 | 0.908 | 0.142 | <0.001 |
|  | **Log_10_(sd_slope)** | -0.680 | 1.775 | 0.696 | 0.258 | <0.001 |
