# Supplementary material for: Estimating home-range size: when to include a third dimension?
Source: Ecol Evol. 2013 Jun 8;3(7):2285–95. doi: 10.1002/ece3.590 (PMC3728965; doi:10.1002/ece3.590)
Supplement: Supplementary file 2 [file ece30003-2285-SD2.doc]

**Appendix S2**. Results of the t-tests applied to the *DIF* values of home-range estimates for areas with altitudinal ranges beneath and above 1800m.

| **Area (km^2^)** | **Altitudinal range (m)** | | **t-value** | **DF** | **p-value** |
| --- | --- | --- | --- | --- | --- |
|  | **<1800** | **≥1800** |  |  |  |
| **100** | 1.30 ± 0.46 | 6.03 ± 4.00 | 16.61 | 391.00 | 0.00 |
| **25** | 1.30 ± 0.46 | 5.98 ± 4.12 | 15.94 | 398.00 | 0.00 |
| **4** | 1.30 ± 0.46 | 6.02 ± 4.43 | 14.98 | 398.00 | 0.00 |
| **1** | 1.30 ± 0.46 | 5.94 ± 4.75 | 13.76 | 398.00 | 0.00 |
| **0.25** | 1.30 ± 0.46 | 5.74 ± 4.84 | 12.90 | 398.00 | 0.00 |
